# Relationship between Microstructure and Properties of Cu–Cr–Ag Alloy

**DOI:** 10.3390/ma13030732

**Published:** 2020-02-06

**Authors:** Dong Liang, Xujun Mi, Lijun Peng, Haofeng Xie, Guojie Huang, Zhen Yang

**Affiliations:** 1 State Key laboratory of Nonferrous Metals and Processes, GRINM Group Co., Ltd, Beijing 100088, China; Daniel.liang@copperalliance.asia (D.L.); penglijun198677@163.com (L.P.); xiehaofeng@grinm.com (H.X.); huangguojie@grinm.com (G.H.); yangzhen365k@163.com (Z.Y.); 2 GRIMAT Engineering Institute Co., Ltd., Beijing 101407, China; 3 General Research Institute for Nonferrous Metals, Beijing 100088, China

**Keywords:** Cu–Cr–Ag alloy, continuous extrusion, aging process, microstructure, physical properties

## Abstract

The microstructure evolution and properties of a Cu–Cr–Ag alloy during continuous extrusion and an aging process were studied by Scanning Electron Microscope (SEM) and Transmission Electron Microscope (TEM). Owing to strong shear deformation that happened during continuous extrusion with working temperatures of 450 to 480 °C, a larger number of fine grains were obtained. Both face-centered cubic (FCC) and body-centered cubic (BCC) precipitates simultaneously existed in the matrix when aged for 450 °C for 2 h, and the Cr phases with BCC structure had an N–W relationship with the matrix. After continuous extrusion, 60% cold deformation, 875 °C × 1 h solid solution treatment, 60% cold deformation, 450 °C × 2 h aging treatment, and 70% cold deformation, the Cu–Cr–Ag alloy acquired excellent comprehensive properties: tensile strength of 494.4 MPa, yield strength of 487.6 MPa, and electrical conductivity of 91.4% IACS.

## 1. Introduction

The Cu–Cr alloy is a typical alloy strengthened through solid solution and aging treatment. Dispersed nanoscale Cr-rich phases precipitate during treatment, which enables the alloy to have high strength, high electrical conductivity, and excellent thermal conductivity. Therefore, the alloy is widely used to manufacture integrated circuit lead frames [1,2,3,4,5,6,7,8,9,10,11], railway contact wires [2,3,4,5,12], resistance welding electrodes [13], and continuous casting machine crystallizer copper plates [14]. It is the focus of R&D efforts in various countries to meet the demand for high-performance copper alloys in high-tech fields such as electricity, metallurgy, aerospace, and atomic energy. So far, the aging-precipitation behavior of the Cu–Cr alloy, and the effect of related elements on the microstructure and properties of the alloy were systematically studied by many scholars, and some research results were obtained [15,16,17,18,19]. However, because the Cr element is easy to oxidize and segregate in a nonvacuum environment, casting by means of traditional vacuum smelting cannot meet the demand for large-length, uniform-performance strip products.

In essence, updrawn continuous casting is an upward continuous casting process, in which the metal melt is pressured into a vacuum crystallizer to continuously solidify. Due to the small size of the crystallizer, the rod blank cools rapidly during solidification, and alloy elements are not easy to segregate. This technology was successfully applied to oxygen-free copper, a Cu–Ag alloy, and some other alloys [20,21,22]. Research shows that large plastic deformation with working temperature results in ultrafine grains, which make the alloy have higher strength while maintaining good conductivity. As a large plastic-deformation-process method, continuous extrusion has been extensively used in industrialized production of aluminum, copper, and other alloys because of its advantages of low energy consumption and continuous production [23,24,25]. To meet the needs for large-length, uniform-performance strip products, Cu–Cr–Ag alloys are prepared by continuous updrawn casting, continuous extrusion, and cold-rolled and aging treatment. In this paper, we investigated the microstructure evolution and properties of a Cu–Cr–Ag alloy during a continuous-manufacturing process to provide detailed experiment data and a theoretical basis for the industrialized production of this alloy. 

## 2. Materials and Methods

**Figure 1:**
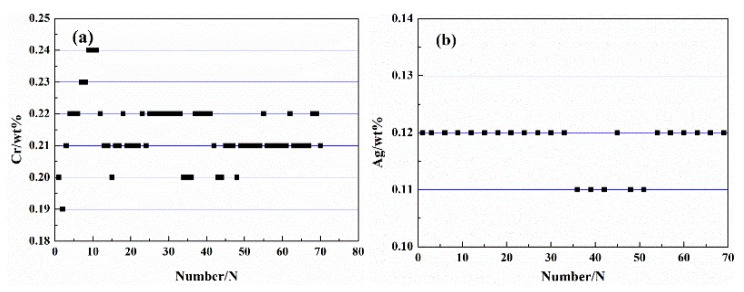
Test results of Cr (**a**) and Ag (**b**) contents in Cu-Cr-Ag alloy (Points at an interval of 7 m or 14 m).

The experimental Cu–Cr–Ag alloy was prepared with electrolytic copper, pure chromium, and pure silver in a continuous furnace under nonvacuum conditions, and then continuously cast in a ceramic rod-shaped mold with a diameter of 20 mm. The corresponding average casting temperature and speed were 1250 °C and 250 mm/min, respectively. The drawn Cu–Cr–Ag was tested for Cr content at 7 meters intervals and Ag content at 14 meters intervals; results are shown in Figure 1. As can be seen from Figure 1, the Cr content of updrawn continuously cast Cu–Cr–Ag alloy was relatively stable at 0.21 ± 0.02 wt %, and the Ag content was stable at 0.11 wt %. Rod blanks of the updrawn continuously cast Cu–Cr–Ag alloy were extruded from a TJ400 Conform continuous extruder to obtain slabs with 12 mm thickness and 40 mm width. During continuous extrusion, the preheating temperature of the die was 450 to 480 °C, and the rotating speed of the extrusion wheel was 7 r/min. The extruded blank was successively subjected to a 60% cold deformation, 875 °C × 1 h solid solution treatment, 60% cold deformation, and aging treatments at 450 and 500 °C for different times. Then, the specimens aged at 450 °C for 2 h were subjected to 50% and 70% cold deformation, respectively. 

The hardness, strength, and electrical conductivity of the alloy specimens in different states were tested using HXD-1000 digital Vickers hardness tester (Wuxi, China), YHS-229WJ universal stretcher (Shanghai, China), and 7501 eddy-current conductivity tester (Xian, China), respectively. The specimens were made into metallographic specimens using a metallographic prototyping machine and a metallographic mosaic machine. After being sanded with sandpaper of different grades and polished, the specimens were corroded by an aqueous solution of FeCl_3_ and HCl in a certain proportion, and the microstructure was observed with a Quanta 200F field emission-environment scanning electron microscope (Hillsboro, America). The average size of the grain was measured by the metallographic and the electron backscatter diffraction method according to the National Standards of P. R, GB/T 36165-2018, and GB/T 6394-2017, respectively.

The microstructures of different states were observed under a JEM 2100 LaB6 transmission electron microscope (Tokyo, Japan). The specimens were prepared through the following steps: we used sandpaper to manually reduce specimen thickness to 50 ± 5 μm for punching, and then placed the specimens on a double-jet thinner for thinning and perforation, where the double-jet thinner was a mixed solution of nitric acid and methanol with a volume ratio of 1:4, and working temperature was −40 to −50 °C.

## 3. Results and Discussion

### 3.1. Microstructure Observation

**Figure 2:**
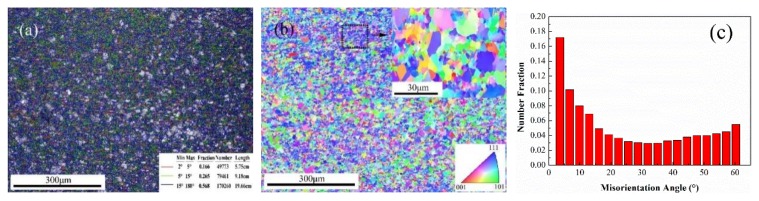
EBSD (Electron Back-Scattered Diffraction) and orientation images of updrawn continuously cast Cu–Cr–Ag alloy blank after continuous extrusion. (**a**) grain boundary distribution map; (**b**) crystallographic orientation map; (**c**) grain size distribution map.

Figure 2 shows the microstructure of an updrawn continuously cast Cu–Cr–Ag alloy blank after continuous extrusion. Figure 2a,b shows that the Cu–Cr–Ag alloy rod black underwent strong shear deformation during the continuous-extrusion process with a working temperature, and the crystal grains were obviously broken, with an average grain size of 4–5 μm. As shown in Figure 2a,c, the broken grains were mainly composed of a large number of large-angle recrystallized grains and few of small-angle subgrains.

**Figure 3:**
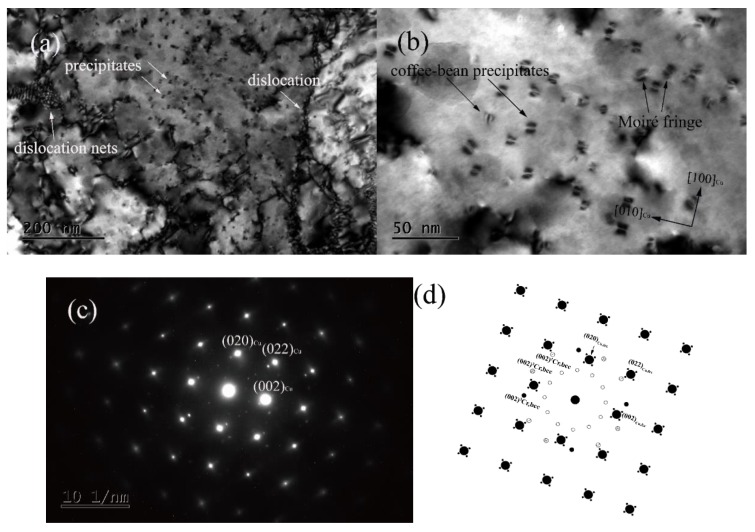
Transmission Electron Microscope (TEM) bright-field image and selected-area diffraction pattern of Cu–Cr–Ag alloy after solid-solution (875 °C × 1 h), 60% cold-deformation, and aging (450 °C × 2 h) treatment. (**a**,**b**) Bright filed image with zone axis of (001)_Cu_; (**c**) SAED pattern of (**b**) with zone axis of (001)_Cu_; (**d**) schematic diagram of (**c**).

Figure 3 shows the Transmission Electron Microscope (TEM) bright-field image and selected-area diffraction pattern of the Cu–Cr–Ag alloy after solution treatment at 875 °C for 1 h, and 60% cold deformation and aging at 450 °C for 2 h. It can be seen from Figure 3a that a large number of dislocations appeared in the matrix and the dislocations interacted with each other. Fine dispersed nanoscale precipitated phases were also found in the matrix. It can be seen in Figure 3b that the size of the precipitated phases was 5–10 nm, and the morphology of the precipitated phases was mainly coffee-bean-shaped and Moire fringe precipitates. The former was mainly distributed in the <100>_Cu_ direction of the matrix, and the latter were distributed in the <110>_Cu_ direction. Figure 3c,d shows the selected-area electron diffraction pattern and schematic diagram along the [001]_Cu_ crystal zone axis, respectively. According to the characteristics of the selected-area electron diffraction pattern shown in Figure 3c,d, two groups of satellite spots appeared around the main diffraction spot, and diffraction stripes appeared in the <011>_Cu_ direction. At the same time, some diffraction spots with relatively weak diffraction brightness were also found, which were mainly distributed along the <011>_Cu_ direction at an angle of 0°, 30°, or 60°. By measuring the crystal-plane spacing of diffraction spots and according to the Bragg equation, diffraction spots near matrix {022}_Cu_ could be regarded as Cr phases with an orderly face-centered cubic (FCC) or a body-centered cubic (BCC) structure. However, it can be seen from the bright-field image in Figure 3a that the Moire fringe precipitates were mainly distributed along the <011>_Cu_ direction of the matrix, which was consistent with the distribution of diffraction spots. In addition, according to Peng et al. [15], the Moire fringe Cr phase had a BCC structure. Therefore, the diffraction spot in this state was defined as the (002) plane of the BCC Cr phase. In addition, the diffraction spots in the <011> _Cu_ direction at an angle of 0°, 30°, or 60° were mainly caused by electron-beam scattering along the (022) plane of the BCC Cr phase. Judging from the diffraction directions of the BCC Cr phase and the matrix, the Cr phases with a bcc structure had an N–W relationship with the matrix. In addition, a circle of diffraction spots appeared between matrix spot and center spot. This group of diffraction spots may have been superlattice spots caused by secondary diffraction of the matrix-diffraction spots, or by the (002) plane of the BCC Cr phase.

### 3.2. Physical Properties

**Figure 4:**
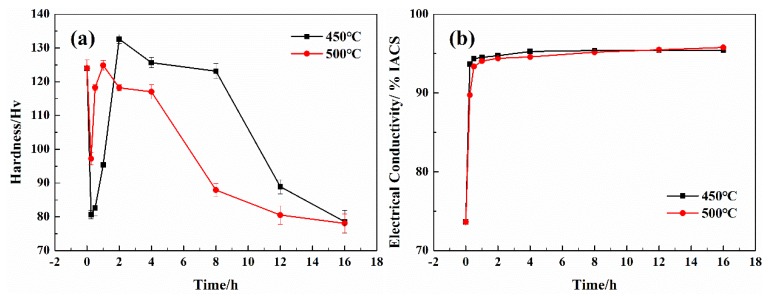
Effect of aging treatment on hardness and electrical conductivity of cold-rolled Cu–Cr–Ag alloy. (**a**) Hardness; (**b**) Electrical conductivity.

Figure 4 shows the effect the aging treatment on the hardness and electrical conductivity of cold-rolled Cu–Cr–Ag alloy. As can be seen from Figure 4a, the hardness of the cold-rolled Cu–Cr–Ag alloy decreased significantly after aging at 450 °C for 15 min, then increased with the extension of aging time. When aging time reached 2 h, the hardness reached its peak value, a Vickers hardness of 132.6. With prolonged aging time, hardness decreased because the alloy was overaged. When the alloy was aged at 500 °C, the relationship between hardness and aging time was the same as that when it was aged at 450 °C, but the time for the alloy to reach peak aging was 1 h, and its peak Vickers hardness was 126.3. That is, when the alloy was aged at 450 and 500 °C, respectively, the relationship curves between Vickers hardness and aging time showed a trend of first descending, then ascending, and then descending again. The excellent mechanical properties of the Cu–Cr–Ag alloy were mainly attributed to a lot of volume fraction of nanoscaled precipitates that were uniformly dispersed in the matrix during the solution annealing and aging process [26,27]. The yield strength of the alloy depended on three different hardening mechanisms: solid-solution strengthening, precipitation hardening, and dislocation strength [28,29]. Owing to the low solid solution of Cr and Ag in the alloy, solid-solution strengthening could be neglected. Therefore, the strength of the alloy was mainly due to precipitation hardening and strain strengthening. Because the alloy underwent a large degree of cold deformation (60%) before aging, a large amount of energy was generated in the alloy. Because the strengthening effect of Cr precipitation was weaker than the lowering of alloy hardness from recrystallization at the early-stage aging process, this resulted in a decrease in the hardness of the Cu–Cr–Ag alloy. With the extension of aging time, many precipitates appeared in the matrix, and their hardening effect was greater than the effect of recovery recrystallization on hardness, leading to an increase in alloy hardness. When aging time exceeded 2 h, the precipitated phase coarsened, its ability to block dislocation decreased, and the precipitation hardening effect gradually weakened, resulting in a decrease in the hardness of the alloy.

It can be seen from Figure 4b that the relationship curves between alloy electrical conductivity and aging time rose sharply at first and then tended to be flat, when the alloy was aged at 450 and 500 °C for different aging times, respectively. This is mainly because the alloy underwent two processes, recovery recrystallization and supersaturated solid-solution precipitation, during the aging process. Complicated microstructure evolution happened during the aging process. The dislocations underwent rearrangement, merger, and disappearance, and a large number of dislocations were converted into subcrystal or large-angle recrystallized structures, which made for the increase in alloy conductivity. Meanwhile, a large amount of Cr alloy elements were precipitated from the matrix. As a result, the effect of the Cr alloy elements and defects on electron scattering was greatly reduced, and alloy conductivity was greatly improved. With the extension of aging time, however, the precipitation kinetics of the alloy elements gradually decreased, and the conductivity of the alloy tended to be stable. 

**Figure 5:**
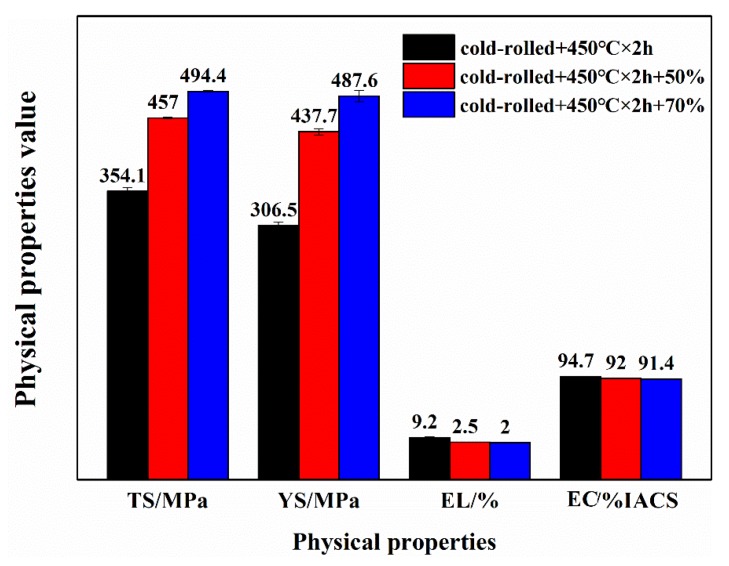
Effect of cold deformation on mechanical and electrical properties of peak aged Cu–Cr–Ag alloy: (TS, Tensile Strength; YS, Yield Strength; EL, Elongation; EC, Electrical Conductivity).

Figure 5 shows the effect of cold deformation on the mechanical and electrical properties of peak-aged Cu–Cr–Ag alloy. With the increase of deformation, the tensile and yield strengths of the alloy gradually increased, while electrical conductivity and elongation gradually decreased, as shown in Figure 5. When the amount of deformation reached 50%, the tensile and yield strengths of the alloy increased by 100 and 130 MPa respectively, while conductivity and elongation decreased by 7% and 2.7%, respectively. When the amount of deformation reached 70%, the tensile and yield strengths of the alloy increased by about 40 and 50 MPa, respectively, while the corresponding elongation and conductivity decreased by 0.5% and 0.6%, respectively. A large number of dispersed nanoscale Cr phases and a small amount of a recrystallized structure were observed in the alloy after being aged at 450 °C for 2 h. When the alloy underwent cold deformation, a fibrous structure was observed, and dislocation density significantly enhanced. The strengthening effect was enhanced through hinder-dislocation movement by the Orowan bypass mechanism. With the increase of deformation, the interaction between precipitated phase and dislocations was further enhanced, but the increase of dislocation density and alloy strength was smaller, and the decrease of alloy conductivity was also smaller.

**Figure 6:**
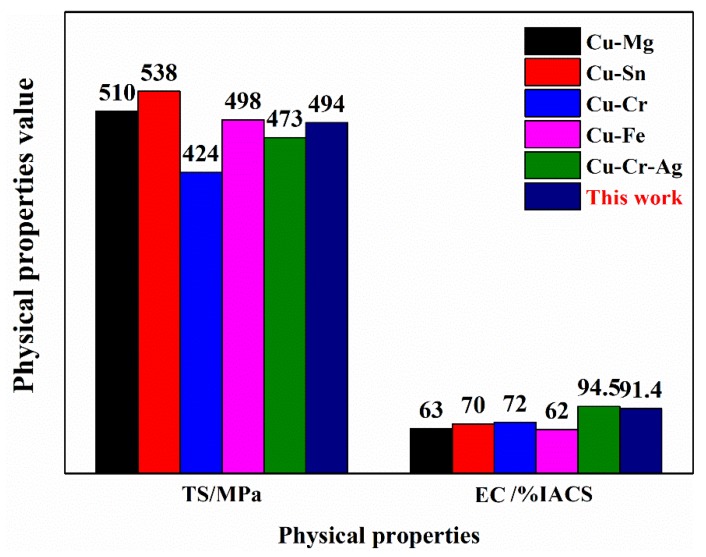
Physical properties of several commonly used high-performance copper alloys (TS, Tensile Strength; EC, Electrical Conductivity).

Figure 6 shows the physical properties of several commonly used high-performance copper alloys. After continuous extrusion, 60% cold deformation, 875 °C × 1 h solid solution treatment, 60% cold deformation, 450 °C × 2 h aging treatment, and 70% cold deformation, the updrawn cast Cu–C–Ag alloy acquired tensile strength of 494.4 MPa and electrical conductivity of 91.4% IACS, having similar tensile strength to but higher electrical conductivity than ordinary Cu–Mg, Cu–Sn, Cu–Cr, and Cu–Fe alloys [30,31,32,33,34,35]. However, compared with the traditional semicontinuous-casting and hot-rolling method, the continuous-manufacturing method is discussed in this paper, which enabled the Cu–Cr–Ag alloy to have higher tensile strength in spite of similar electrical conductivity.

## 4. Conclusions

(1) With the combination of strong shear deformation and working temperature, the crystal grains of the updrawn cast Cu–Cr–Ag alloy were obviously broken with an average grain size of 4–5 μm during continuous extrusion.
(2) A large number of nanoscale coffee-bean-shaped and Moire fringe Cr phases were observed aged at 450 °C for 2 h. The Moire fringe Cr phases had an N–W relationship with the matrix.
(3) After continuous extrusion, 60% cold deformation, 875 °C × 1 h solid solution treatment, 60% cold deformation, 450 °C × 2 h aging treatment, and 70% cold deformation, the updrawn cast Cu–Cr–Ag alloy acquired excellent comprehensive properties represented by tensile strength of 494.4 MPa, yield strength of 487.6 MPa, and electrical conductivity of 91.4 % IACS.

## References

[1] Hatakeyama M., Toyama T., Nagai Y., Hasegawa M., Eldrup M., Singh B.N. (2008). Nanostructural evolution of Cr-rich precipitates in a Cu-Cr-Zr alloy during heat treatment studied by 3 dimensional atom probe. Mater. Trans..

[2] Cheng J., Shen B., Yu F. (2013). Precipitation in a Cu–Cr–Zr–Mg alloy during aging. Mater. Character..

[3] Zhou J., Zhu D., Tang L., Jiang X., Chen S., Peng X., Hu C. (2016). Microstructure and properties of powder metallurgy Cu–1% Cr–0.65% Zr alloy prepared by hot pressing. Vacuum.

[4] Liu Q., Zhang X., Ge Y., Wang J., Cui J.-Z. (2006). Effect of processing and heat treatment on behavior of Cu-Cr-Zr alloys to railway contact wire. Metall. Mater. Trans. A.

[5] Pang Y., Xia C., Wang M., Li Z., Xiao Z., Wei H., Sheng X., Jia Y., Chen C. (2014). Effects of Zr and (Ni, Si) additions on properties and microstructure of Cu–Cralloy. J. Alloys Compd..

[6] Xia C., Zhang W., Kang Z., Jia Y., Wu Y., Zhang R., Xu G., Wang M. (2012). High strength and high electrical conductivity Cu–Cr system alloys manufactured by hot rolling–quenching process and thermomechanical treatments. Mater. Sci. Eng. A.

[7] Wang Z., Zhong Y., Cao G., Wang C., Wang J., Ren W., Lei Z., Ren Z. (2009). Influence of dc electric current on the hardness of thermally aged Cu–Cr–Zr alloy. J. Alloys Compd..

[8] Fuxiang H., Jusheng M., Honglong N., Zhiting G., Chao L., Shumei G., Xuetao Y., Tao W., Hong L., Huafen L. (2003). Analysis of phases in a Cu-Cr-Zr alloy. Scr. Mater..

[9] Cheng J., Yu F., Shen B. (2014). Solute clusters and chemistry in a Cu–Cr–Zr–Mg alloy during the early stage of aging. Mater. Lett..

[10] Zhang Y., Volinsky A.A., Tran H.T., Chai Z., Liu P., Tian B., Liu Y. (2007). Aging behavior and precipitates analysis of the Cu–Cr–Zr–Ce alloy. J. Mater. Sci. Technol..

[11] Correia J., Davies H., Sellars C. (1997). Sellars. Strengthening in rapidly solidified age hardened Cu–Cr and Cu–Cr–Zr alloys. Acta Mater..

[12] Chbihi A., Sauvage X., Blavette D. (2012). Atomic scale investigation of Cr precipitation in copper. Acta. Mater..

[13] Hatakeyama M., Toyama T., Yang J., Nagai Y., Hasegawa M., Ohkubo T., Eldrup M., Singh B.N. (2009). 3D-AP and positron annihilation study of precipitation behavior in Cu-Cr-Zr alloy. Nucl. Mater..

[14] Jin Y., Adachi K., Takeuchi T., Suzuki H. (1998). Ageing characteristics of Cu–Cr in-situ composite. Mater. Sci..

[15] Peng L., Xie H., Huang G., Xu G., Yin X., Feng X., Mi X., Yang Z. (2017). The phase transformation and strengthening of a Cu-0.71 wt% Cr alloy. J. Alloys Compd..

[16] Batra I., Dey G., Kulkarni U., Banerjee S. (2002). Precipitation in a Cu–Cr–Zr alloy. Mater. Sci. Eng. A.

[17] Zhao Z., Xiao Z., Li Z., Ma M., Dai J. (2018). Effect of magnesium on microstructure and properties of Cu-Cr alloy. J. Alloys Compd..

[18] Watanabe C., Monzen R., Tazaki K. (2008). Mechanical properties of Cu–Cr system alloys with and without Zr and Ag. J. Mate. Sci..

[19] Huaqing L., Shuisheng X., Xujun M., Yong L., Pengyue W., Lei C. (2006). Influence of Cerium and Yttrium on Cu-Cr-Zr Alloys. J. Rare Earths..

[20] Liu Q., Cui Z., Xu G., Liu X. (2004). Experimental study of Cu-Ag alloy contact wire manufactured through upward-casting process. J. Northeast. Uni..

[21] Zhang Z. (2013). Study on High Conductivity and High strength Copper-silver Alloy wire and New Continuously Manufacturing Techniques. Master’s Thesis.

[22] Yuan Y., Li Z., Xiao Z., Zhao Z., Yang Z. (2017). Microstructure evolution and properties of Cu-Cr alloy during continuous extrusion process. J. Alloys Compd..

[23] Sun J., Liu P., Liu X., Chen X., He D., Ma F., Li W. (2014). Microstructure evolution and properties of Cu-Ni-Si alloy during continuous extrusion process CHIN. J. Nonferrous. Met..

[24] Thomas B., Derguti F., Jackson M. (2017). Continuous extrusion of a commercially pure titanium powder via the Conform process. Mater. Sci. Technol..

[25] Yuan Y., Li Z., Xiao Z., Zhao Z. (2017). Investigations on voids formation in Cu–Mg alloy during continuous extrusion. JOM.

[26] Holzwarth U., Stamm H. (2000). The precipitation behaviour of ITER-grade Cu–Cr–Zr alloy after simulating the thermal cycle of hot isostatic pressing. J. Nucl. Mater..

[27] Xie H., Mi X., Huang G., Gao B., Yin X., Li Y. (2011). Effect of thermomechanical treatment on microstructure and properties of Cu-Cr-Zr-Ag alloy. Rare Met..

[28] Krishna S.C., Gangwar N.K., Jha A.K., Pant B., George K.M. (2014). Properties and Strengthening Mechanisms in Cold-Rolled and Aged Cu–3Ag–0.5Zr Alloy. Metal. Micro. Analy.

[29] Freudenberger J., Lyubimova J., Gaganov A., Klauß H., Schultz L. (2010). Mechanical behaviour of heavily deformed CuAgZr conductor materials. J. Phys. Confer.

[30] Ma M., Li Z., Qiu W., Xiao Z., Zhao Z., Jiang Y. (2019). Microstructure and properties of cu–mg-ca alloy processed by equal channel angular pressing. J. Alloys Compd..

[31] Chen L., Zhou B., Han J., Xue Y.Y., Jia F., Zhang X.G. (2013). Effects of alloying and deformation on microstructures and properties of Cu–Mg–Te–Y alloys. Trans. Nonferrous Met. Soc. China.

[32] Yan M., Wu Y., Chen J., Zhou X. (2011). Microstructure Evolution in Preparation of Cu-Sn Contact Wire for High-speed Railway. Adv. Mater. Res..

[33] Dong Q., Shen L., Cao F., Jia Y., Liao K., Wang M. (2015). Effect of thermomechanical processing on the microstructure and properties of a Cu–Fe–P alloy. J. Mater. Eng. Perform..

[34] Shangina D., Maksimenkova Y., Bochvar N., Serebryany V. (2016). Influence of alloying with hafnium on the microstructure, texture, and properties of Cu–Cr alloy after equal channel angular pressing. J. Mater. Sci..

[35] Liu Y., Li Z., Jiang Y., Zhang Y., Zhou Z. (2017). The microstructure evolution and properties of a Cu-Cr-Ag alloy during thermal-mechanical treatment. J. Mater. Res..

